# Supplementary material for: The Use of Mushrooms and Spirulina Algae as Supplements to Prevent Growth Inhibition in a Pre-Clinical Model for an Unbalanced Diet
Source: Nutrients. 2021 Nov 29;13(12):4316. doi: 10.3390/nu13124316 (PMC8705242; doi:10.3390/nu13124316)
Supplement: Supplementary file 1 [file nutrients-13-04316-s001.zip › nutrients-1470558-supplementary.pdf]

Table S1. Growth patterns – body weight.

Day	CON	UBD	UBD+SP	UBD+PE	UBD+AB	UBD+MIX
1	a	a	a	a	a	a
4	a	ab	b	b	ab	b
8	a	b	b	b	b	b
11	a	b	b	b	b	b
15	a	c	bc	bc	bc	b
18	a	c	b	bc	b	b
22	a	c	b	b	b	ab
25	a	c	b	bc	b	ab
29	a	c	b	b	b	ab
32	a	c	b	b	b	ab
36	a	c	b	b	b	ab
39	a	c	b	b	b	ab
43	a	c	b	b	b	ab

Statistical difference of body weight presented in graph 1A according to measurements days.
Different superscript letters are significantly different ($P < 0.05$) by one-way ANOVA followed by Tukey's test.

Table S2. Growth patterns – body length.

Day	CON	UBD	UBD+SP	UBD+PE	UBD+AB	UBD+MIX
1	a	a	a	a	a	a
8	a	a	a	a	a	a
15	a	b	b	b	ab	ab
22	a	c	bc	bc	ab	ab
29	a	c	abc	bc	ab	ab
36	a	c	b	b	Ab	ab
43	a	d	bcd	cd	abc	ab

Statistical difference of body length presented in graph 1B according to measurements days.
Different superscript letters are significantly different ($P < 0.05$) by one-way ANOVA followed by Tukey's test.

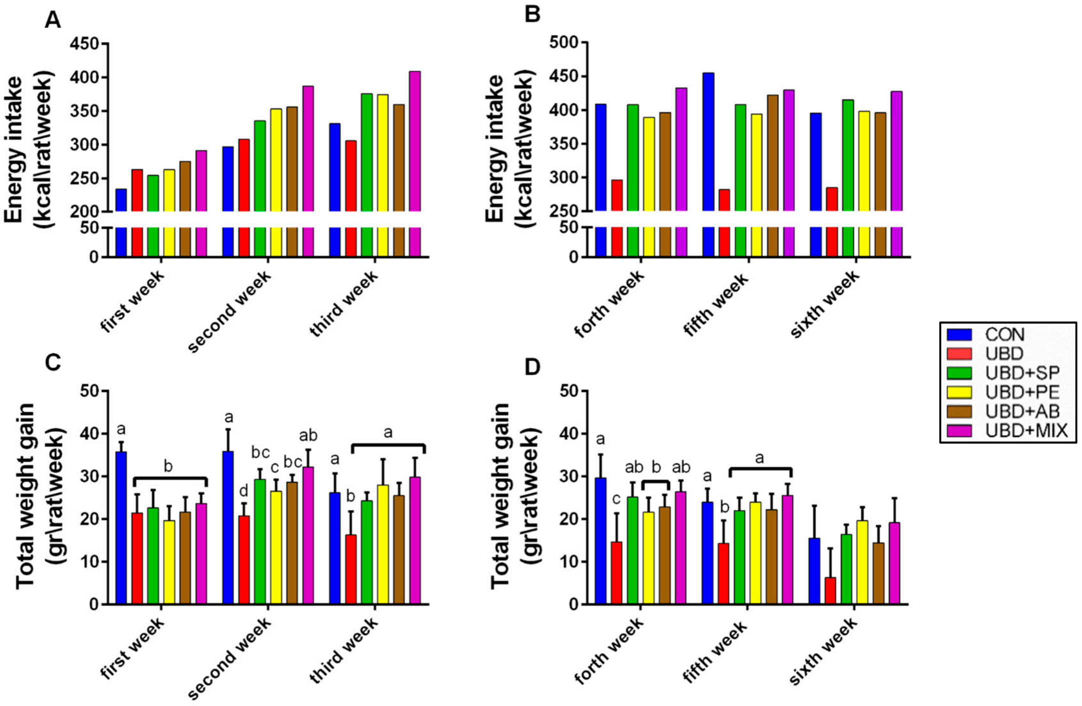

Figure S1. Energy intake and weight gain per week. (**A**,**B**) Energy intake (kcal\rat\week). (**C**,**D**) Total weight gain (gr\rat\week). Values are expressed as mean ± SD of n = 8 rats/group, different superscript letters are significantly different ($p < 0.05$) by one-way ANOVA followed by Tukey's test.

Table S3. Amino acids content in the three supplements.

**Amino acids (gr\kg diet)**		**Supplements**		
		Spirulina algae	Pleurotus eryngii	Agaricus bisporus
**Essential**	Histidine	0.03394	0.01289	0.00980
	Isoleucine	0.10971	0.02368	0.01785
	Leucine	0.15727	0.03491	0.03486
	Lysine	0.07709	0.02242	0.00795
	Methionine[a]	0.02783	0.00639	0.00389
	Phenylalanine[b]	0.09785	0.02200	0.02007
	Threonine	0.07791	0.02214	0.01294
	Valine	0.15098	0.02953	0.02245
	Tryptophan	ND	ND	ND
**Conditionally essential**	Tyrosine[b]	0.05440	0.00978	0.00920
	Cystine[a]	0.03140	0.00674	0.00504
	Arginine	0.10613	0.02202	0.01276
**Non-essential**	Alanine	0.13362	0.03344	0.02220
	Aspartic acid	0.10991	0.02871	0.02009
	Glutamic acid	0.25057	0.08829	0.09119
	Glycine	0.09314	0.02213	0.01990
	Proline	0.06888	0.02286	0.03090
	Serine	0.06018	0.01800	0.01583
	Asparagin	ND	ND	ND
	4-Aminobutyric acid	0.00027	0.00357	0.00126

LC-MS/MS analysis shows the amino acids content in the powders. Values are presented as the addition of amino acids from each supplement per kg diet.

ND =not detected.
[a]One-half of L-methionine may be replaced by L-cystine
[b]One-half of L-phenylalanine may be replaced by L-tyrosine

Table S4. % Amino acids per kg diet from the NRC.

**%Amino acid/ NRC**		**Diets**					
		CON	UBD	UBD+SP	UBD+PE	UBD+AB	UBD+MIX
**Essential**	Histidine	192.86	96.43	97.64	96.89	96.78	98.45
	Isoleucine	190.32	95.16	96.93	95.54	95.45	97.6
	Leucine	168.22	84.11	85.58	84.44	84.44	86.23
	Lysine	158.70	79.35	80.19	79.59	79.43	80.52
	Methionine	89.23	44.62	45.04	44.71	44.68	45.2
	Phenylalanine	152.94	76.47	77.91	76.79	76.77	78.53
	Threonine	129.03	64.52	65.77	64.87	64.72	66.34
	Valine	186.49	93.24	95.28	93.64	93.55	95.99
	Tryptophan	130.00	65.00	65.00	65.00	65.00	65.00
**Conditionally essential**	Tyrosine	329.41	**164.71**	**166.31**	**164.99**	**164.98**	**166.86**
	Cystine	218.18	**109.09**	**110.04**	**109.3**	**109.24**	**110.4**
	Arginine	172.09	86.05	88.51	86.56	86.34	89.32
**Non-essential**	Alanine	140	70	73.34	70.84	70.55	74.73
	Aspartic acid	290	**145**	**147.75**	**145.72**	**145.5**	**148.97**
	Glutamic acid	106	53	53.38	52.97	52.98	53.83
	Glycine	63	32	33.22	32.04	32	33.92
	Proline	550	**275**	**276.72**	**275.54**	**275.77**	**278.07**
	Serine	280	**140**	**141.5**	**140.45**	**140.4**	**142.35**
	Asparagin						
	4-Aminobutyric acid						

Values are presented as % amino acids per kg diet from the NRC 1994 (4th rev.) recommendations. Bold values represent reaching to the minimum necessary in the unbalance diets according to the NRC.

Table S5. Minerals content in the three supplements.

Minerals (mg\kg diet)		**Supplements**		
		**Spirulina algae**	**Pleurotus eryngii**	**Agaricus bisporus**
**Essential mineral elements**	Calcium	12.95	2.457	4.330
	Phosphorus	38.93	38.273	18.277
	Potassium	73.62	99.656	21.655
	Sulfur	-	-	-
	Sodium	51.44	0.317	0.283
	Chloride	50.25	3.556	2.793
	Magnesium	14.21	5.544	5.471
	Iron	2.145	0.858	3.969
	Zinc	0.071	0.282	0.184
	Manganese	0.134	0.070	0.099
	Copper	0.014	0.055	0.024
	Iodin	-	-	-
	Molybdenum	0.0012	0.0006	0.0045
	Selenium	<0.002	<0.0014	<0.0014
**Potentially beneficial mineral elements**	Silicon	-	-	-
	Chromium	0.0016	0.0100	0.0104
	Fluoride	-	-	-
	Nickel	0.0034	0.0056	0.0061

	Boron	-	-	-
	Lithium	-	-	-
	Vanadium	-	-	-
	Lead	<0.0003	0.0009	0.0011
	Tin	0.0007	0.00016	0.0002
	Cadmium	<0.00015	0.0006	0.0001
	Mercury	<0.00065	<0.00046	<0.00046
	Antimony	<0.00015	<0.00011	<0.00011

ICP-OES and ICP-MS analyses shows the mineral content in the powders. Values are presented as the addition of minerals from each supplement per kg diet

Table S6. % Minerals per kg diet from the NRC.

**%Minerals/ NRC**		**Diets**					
		CON	UBD	UBD+SP	UBD+PE	UBD+AB	UBD+MIX
**Essential mineral elements**	Calcium	100	50	50.259	50.049	50.087	50.395
	Phosphorus	52.033	26.017	27.314	27.292	26.626	29.199
	Potassium	100	50	52.045	52.768	50.602	55.415
	Sulfur						
	Sodium	203.800	**101.9**	**112.188**	**101.963**	**101.957**	**112.308**
	Chloride	314.200	**157.100**	**167.150**	**157.811**	**157.659**	**168.420**
	Magnesium	101.400	50.700	53.542	51.809	51.794	55.745
	Iron	100	50	56.129	52.45	61.34	69.919
	Zinc	250	**125**	**125.588**	**127.351**	**126.534**	**129.473**
	Manganese	100	50	51.335	50.704	50.991	53.029
	Copper	120	60	60.283	61.098	60.476	61.857
	Iodin	133.333	66.667	66.667	66.667	66.667	66.667
	Molybdenum	100	50	50.787	50.425	52.973	54.184
	Selenium	100	50	51.333	50.933	50.933	53.2

Values are presented as % minerals per kg diet from the NRC 1994 (4th rev.) recommendations. Bold values represent reaching to the minimum necessary according to the NRC.
